# Characterization and phylogenetic analysis of the complete chloroplast genome sequence of *Disanthus cercidifolius* subsp. *longipes* (Hamamelidaceae), a rare and endangered wild plant species in China

**DOI:** 10.1080/23802359.2020.1731372

**Published:** 2020-02-28

**Authors:** Ming Jiang, Junfeng Wang, Huijuan Zhang

**Affiliations:** a Zhejiang Provincial Key Laboratory of Plant Evolutionary and Conservation, College of Life Science Taizhou University, Jiaojiang, Zhejiang, China;; b Lishui Institute of Forestry, Lishui, Zhejiang, China

**Keywords:** *Disanthus cercidifolius* subsp. *longipes*, chloroplast genome, phylogenetic analysis, rare plant species

## Abstract

*Disanthus cercidifolius* subsp. *longipes* is a rare and endangered plant species. In our study, the complete chloroplast genome was assembled by using high-throughput DNA sequencing data. The whole CP genome is 158,076 bp in length, comprising of a large single-copy region of 87,148 bp, a small single-copy region of 18,300 bp, and two inverted repeat regions of 26,314 bp each. There are 136 genes in the genome, including 86 protein-coding genes, 40 transfer RNA genes, eight ribosomal RNA genes, and two pseudogenes (*ndhK* and *ycf1*). Phylogenetic results demonstrated that *D. cercidifolius* subsp. *longipes* grouped with other Hamamelidaceae species, with a support rate of 100%.

The genus *Disanthus* belongs to Hamamelidaceae family, and it is a monotypic genus with a plant species of *Disanthus cercidifolius* (Wu et al. 2003; Gao et al. 2009). *Disanthus cercidifolius* subsp. *longipes* is a subspecies of *D. cercidifolius* in China–Japan flora region (Yu et al. 2014). This plant is a shrub growing to about four meters, with brown branchlets, broadly ovate-rounded leaves, and red petals. *D. cercidifolius* subsp. *longipes* is listed as an endangered plant species by the IUCN, and in China, it distributes only in provinces of Hunan, Jiangxi, and Zhejiang, with a small number of individuals. In Zhejiang province, the species is now listed as a key protected wild plant. In this study, we assembled and annotated the complete chloroplast (CP) genome of *D. cercidifolius* subsp. *longipes* to understand its features as well as its phylogenetic relationship with other plant species.

Fresh leaves were sampled in Longquanshan (27°53′42′′N, 119°10′11′′E), Longquan, Zhejiang Province, and they were kept in plastic bags before taking to the laboratory. A voucher specimen (CHS2018012) was deposited in the Molecular Biology Laboratory of Taizhou University. Total genomic DNA was isolated according to the CTAB protocol described by Doyle and Doyle (1987). A DNA library was prepared following the protocol supplied by Illumina Inc. (San Diego, CA) and was then sequenced using an Illumina Hiseq X Ten system. Approximately 6.3 GB of 150 bp paired-end reads were generated, and they were filtered by NGS QC Toolkit v2.3.3 to trim off adapters and remove low quality reads (Patel and Jain 2012). The CP genome was then assembled by running the Perl program in NOVOPlasty package (Dierckxsens et al. 2017), and it was annotated using DOGMA (Wyman et al. 2004). The complete plastome (GenBank accession: MN527332) is 158,076 bp in length with a typical quadripartite structure. The sizes of large single-copy region (LSC), small single-copy region (SSC), and inverted repeat regions (IRs) are 87,148, 18,300, and 26,314 bp, respectively. Totally, 136 genes are annotated in the CP genome, these include 86 protein-coding genes, 40 transfer RNA genes, eight ribosomal RNA genes, and two pseudogenes. Two protein-coding genes, *ndhK* and *ycf1*, were identified as pseudogenes. The overall GC content in the CP genome is 37.9%, while in LSC, SSC, and IR, the GC contents are 36.0, 32.9, and 43.1%, respectively.

To understand the evolutionary relationship between *D. cercidifolius* subsp. *longipes* and related plant species whose CP genomes were assembled, the complete CP genomes of *Chunia bucklandioides* (NC_041163), *Corylopsis coreana* (NC_040141), *Corylopsis spicata* (MK942341), *Sinowilsonia henryi* (MF687003), *Liquidambar formosana* (NC_023092), *Cercidiphyllum japonicum* (NC_037940), *Fortunearia sinensis* (NC_041487), *Rhodoleia championii* (NC_045276), *Parrotia subaequalis* (MG334121), *Hamamelis mollis* (NC_037881), and 11 *Hydrangea* plants were downloaded from NCBI. A maximum-likelihood tree was constructed based on GTR + G+I model with both *Aristolochia contorta* and *Aristolochia debilis* as the outgroup by using PhyML 3.1 (Guindon et al. 2010). The phylogenetic analysis results indicated *D. cercidifolius* subsp. *longipes* and other nine Hamamelidaceae species grouped in the same clade, with a support rate of 100% (Figure 1).

**Figure 1.**
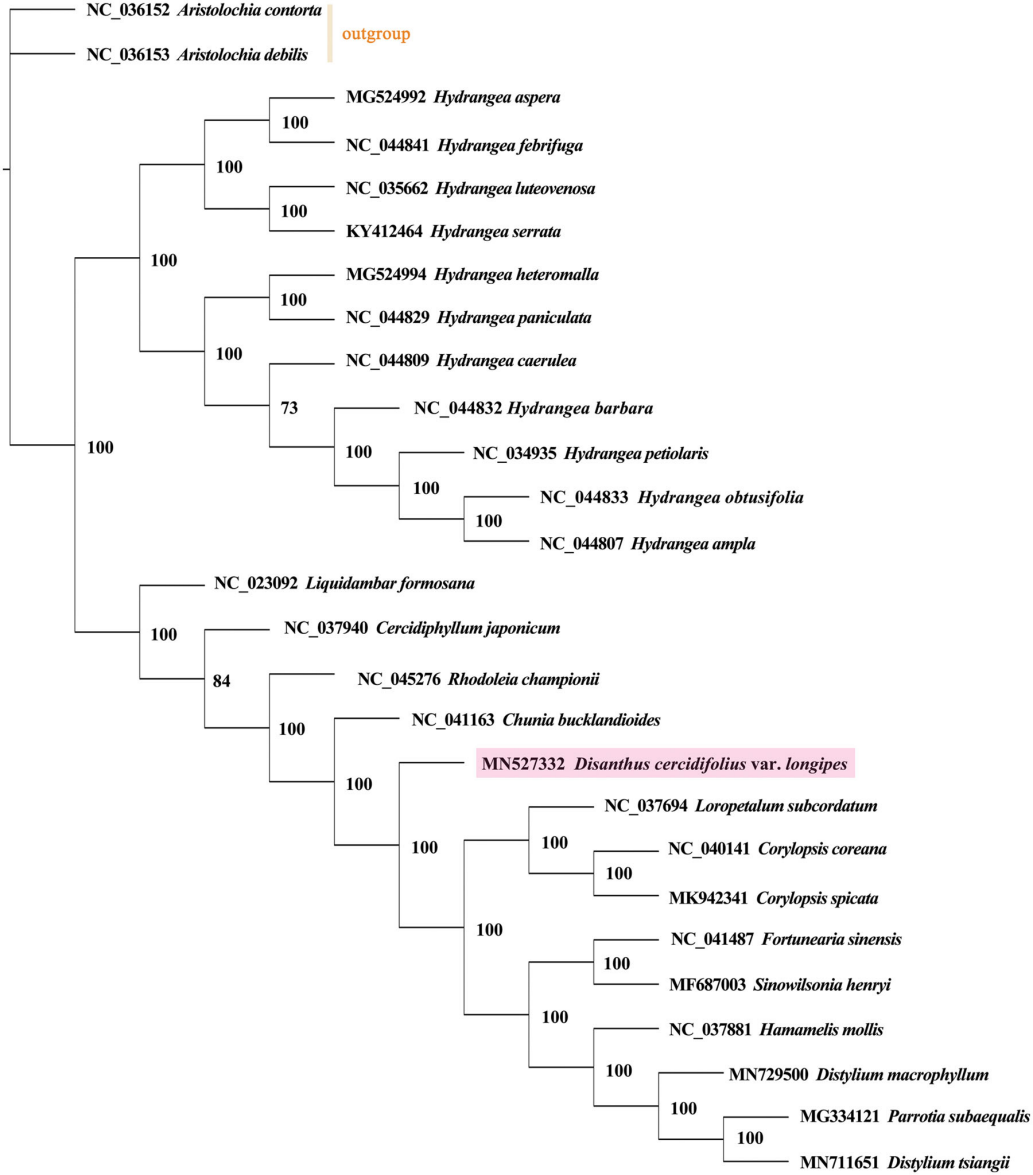
A maximum-likelihood tree based on the complete chloroplast genome sequences of *Disanthus cercidifolius* subsp. *longipes* (Hamamelidaceae) and related species, with both *Aristolochia contorta* and *A*. *debilis* (Aristolochiaceae) as the outgroup. The numbers next to nodes indicate bootstrap support values.
